# Integrating data from multidisciplinary Management of Malignant Pleural Mesothelioma: a cohort study

**DOI:** 10.1186/s12885-021-08532-x

**Published:** 2021-07-01

**Authors:** Laura Saracino, Chandra Bortolotto, Stefano Tomaselli, Elia Fraolini, Matteo Bosio, Giulia Accordino, Francesco Agustoni, David M. Abbott, Emma Pozzi, Dimitrios Eleftheriou, Patrizia Morbini, Pietro Rinaldi, Cristiano Primiceri, Andrea Lancia, Patrizia Comoli, Andrea R. Filippi, Giulia M. Stella

**Affiliations:** 1 Department of Medical Sciences and Infective Diseases, Unit of Respiratory Diseases, IRCCS Policlinico San Matteo Foundation and University of Pavia Medical School, 27100 Pavia, Italy; 2 Department of Intensive Medicine, Unit of Radiology, IRCCS Policlinico San Matteo Foundation and University of Pavia Medical School, Pavia, Italy; 3 Department of Medical Sciences and Infective Diseases, Unit of Oncology, IRCCS Policlinico San Matteo Foundation and University of Pavia Medical School, 27100 Pavia, Italy; 4 Department of Anesthesia and Intensive Care, IRCCS Policlinico San Matteo Foundation and University of Pavia Medical School, 27100 Pavia, Italy; 5 Department of Internal Medicine, ASST, Pavia, Italy; 6 Department of Molecular Medicine, Unit of Pathology, IRCCS Policlinico San Matteo Foundation and University of Pavia Medical School, Pavia, Italy; 7 Department of Intensive Medicine, Unit of Cardiothoracic Surgery, IRCCS Policlinico San Matteo Foundation and University of Pavia Medical School, Pavia, Italy; 8 Department of Medical Sciences and Infective Diseases, Unit of Radiation Therapy, IRCCS Policlinico San Matteo Foundation and University of Pavia Medical School, Pavia, Italy; 9 Cell Factory and Pediatric Hematology-Oncology Unit, IRCCS Fondazione Policlinico San Matteo, Pavia, Italy

**Keywords:** Mesothelioma, Multidisciplinary team, Asbestos, Imaging

## Abstract

**Background:**

Malignant pleural mesothelioma (MPM) is a rare and aggressive malignancy that most commonly affects the pleural layers. MPM has a strong association with asbestos, mainly caused by exposure to its biopersistent fibers in at least 80% of cases. Individuals with a chronic exposure to asbestos might develop disease with a 20–40-year latency with few or no symptoms. Such has been the case in the Italian regions of Piedmont and Lombardy, where industrial production of materials laden with asbestos, mainly cements, has been responsible for the onset of a large epidemic. Since 2018, a multidisciplinary team at San Matteo hospital in Pavia has been collecting data on over 100 patients with MPM. The main goal of this project is to define and describe an integrated profile for each MPM case at diagnosis by using data mining and partition analysis.

**Methods:**

Here we bring together exhaustive epidemiologic, histologic and radiologic data of 88 MPM patients that came to our observation and draw correlations with predictive and prognostic significance.

**Results:**

The median overall survival (OS) was 15.6 months. Most patients presented with pleural effusion, irrespective of disease stage. Quite unexpectedly, no statistically significant association was demonstrated between OS and TNM disease stage at diagnosis. Although average OS is similar in male and female patients, partition analysis of data underlined a significant differential hierarchy of predictor categories based on patient gender. In females with no smoking history, full chemotherapeutic regimens are associated with better outcomes. Moreover, concerning second line treatments, vinorelbine emerged as the most advantageous choice for female patients, whereas in the male subgroup no statistically significant difference resulted between gemcitabine and vinorelbine.

**Conclusion:**

A multidisciplinary approach to MPM is mandatory to define better therapeutic approaches, personalize the management and improve patient outcomes.

**Supplementary Information:**

The online version contains supplementary material available at 10.1186/s12885-021-08532-x.

## Background

Mesothelioma is a rare and aggressive malignant tumor which arises from mesothelial linings, most frequently affecting the pleura (90%), but also the peritoneum, the pericardium, and the tunica vaginalis. Although molecular steps leading to the onset of malignant pleural mesothelioma (MPM) are partially known, the disease is still lacking effective therapies. Novel biological agents (from small molecules to checkpoint inhibitors) although successfully used for the treatment of different epithelial-derived tumors, have shown scarce efficacy against MPM [1–5]. It is well documented that at least 80% of mesothelioma cases are caused by exposure to asbestos [6–8]. In developed countries like Italy, roughly 1.15 per 100,000 cases are diagnosed annually [9, 10]. The Piedmont and Lombardy regions are the most affected, due to industrial production. In the last decade, more than 300 new cases have been diagnosed per year in Lombardy alone. In 2018, the incidence of MM in Italy was 6.08 cases/100,000 inhabitants, 8.48 cases/100,000 males and 4.38 cases/100,000 females [11–13]. Since 2018, our Institution has been dedicated to the organization of an integrated path to MPM diagnostic and therapeutic workup. This approach provides a unique opportunity to analyze patient’s clinical data with a multidisciplinary perspective. More specifically, we here aimed at matching the clinical, pathological, and imaging features of the disease to outline specific patterns predictive of patient outcomes. The main goal of this project is to define and describe an integrated profile for each MPM case at diagnosis by using data mining and partition analysis. Ultimately, this work will provide preliminary findings to develop future omic-centered projects in the MPM population.

## Methods

### The Pavia experience: territorial diagnostic, therapeutic and assistance planning

The PDTA (Percorso Diagnostico Terapeutico e Assistenziale-Territorial Diagnostic, Therapeutic and Assistance Planning) for MPM was first defined in 2014 within the Provincial Oncology Intercompany Department (DIPO) of Pavia. In 2018, the PDTA established two territorial outpatient facilities in view of local epidemiological data on asbestos-related diseases: a first-level clinic, located at the PRESST in the town of Broni (in the province of Pavia), and a second-level clinic managed by the Pneumology Unit and Medical Oncology Unit at the IRCCS Policlinico San Matteo (University hospital, located in Pavia). The two structures cooperate to provide care for patients with suspected asbestos-related pathology. They perform first-level diagnostic investigations as well as those of greater complexity to allow diagnosis and tumor staging. Moreover, they cooperate to provide an individualized therapeutic pathway and psycho-social care plan for the patients. In addition, the PDTA manages the chemotherapy treatments available at our territorial facilities (PRESST Broni, Broni-Stradella Hospital), referring to IRCCS San Matteo when high complexity investigations are needed.

### Patients identification and selection

**Fig. 1:**
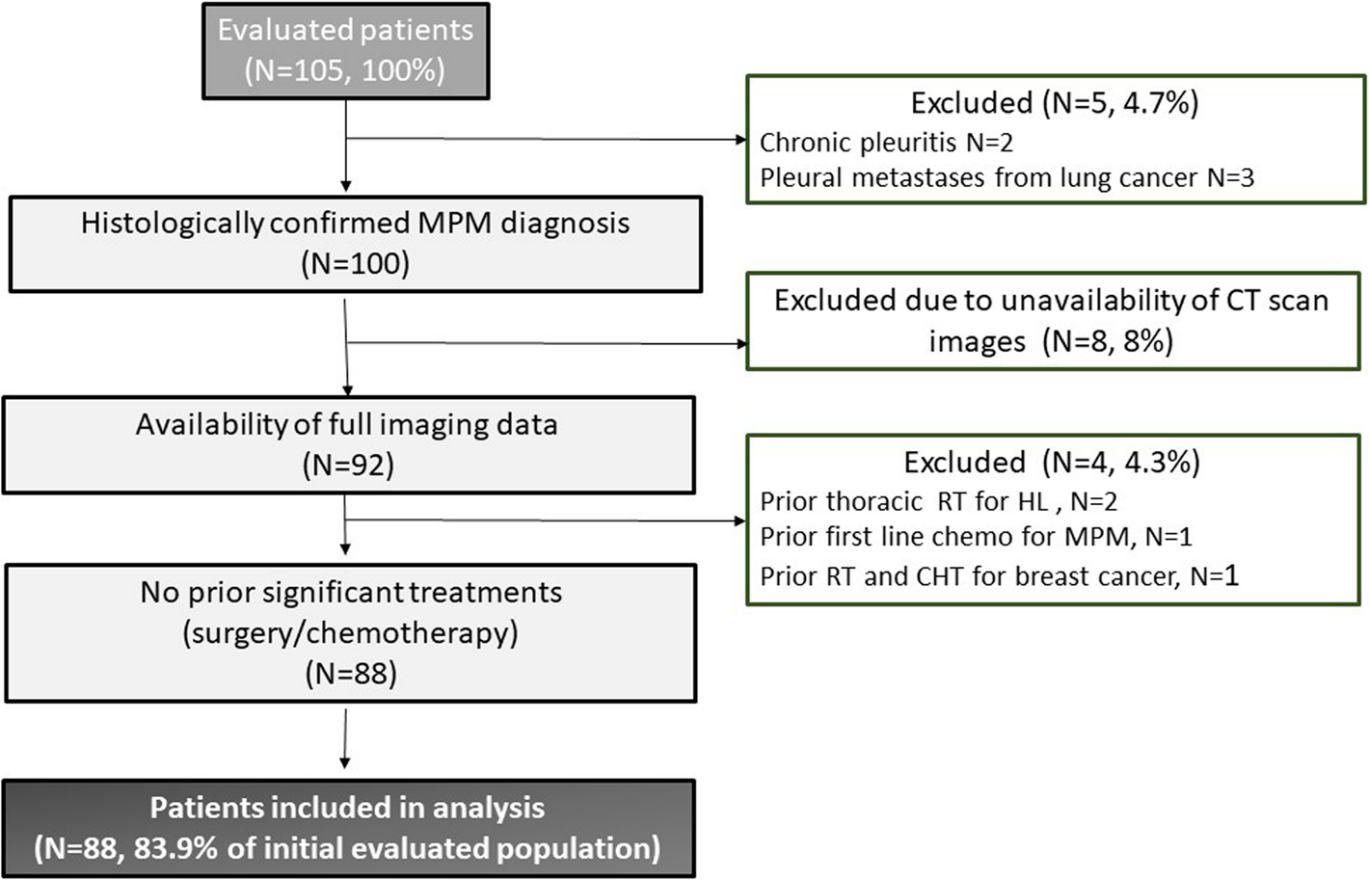
The study population. A total of 105 patients were multidisciplinarly evaluated and entered the PDTA path. We excluded from the initial study population, two only cases who were early enrolled in clinical trials in other Institutions. Based on exclusion criteria,17 patients were not included in the analysis. HL = Hodgkin lymphoma

Since November 2018, we have been collecting data on all mesothelioma patients managed through the PDTA. The number of patients was then narrowed by several criteria to be included in the study, as represented in Fig. 1. Data were obtained from the anamnestic records, outpatient reports and hospital discharge letters. Informed consent of each patients was collected routinely at hospital admission in accordance with standard hospital procedures. Imaging findings were graded by two radiologists in blind; the experienced radiologist was the referring radiologist for the “multidisciplinary board of thoracic neoplasms” while the non-experienced radiologist was a radiology resident with less than 5-year-experience in thoracic radiology. Disagreement between the two radiologists were solved by mutual agreement.

### Statistical analysis

Data collection was made in the form of Excel Spreadsheets. Basic statistical analysis was conducted through the Excel “Data Analysis” add-on package. Survival curves were estimated with Kaplan-Meier method. Advanced statistical analysis of data was performed by using the JMP partition algorithm (JMP-Statistical Discoveries. From SAS, website at www.jmp.com) which is able to search all possible splits of best response predictors. Decision trees are a classification methodology, wherein the classification process is constructed with the use of a set of hierarchical decisions on the feature variables, arranged in a tree-like structure. The decision at a particular node of the tree, which is referred to as the split criterion, is typically a condition on one or more feature variables in the training data. These splits (or partitions) of the data are done recursively in top-down fashion by using the split criteria, through an iterative approach. A small number of features are removed in each iteration, according to the split criteria. Then, the classifier is retrained on the pruned set of features to re-estimate the weights. The decision tree is typically constructed as a hierarchical partitioning of the training examples, just as a top-down clustering algorithm partitions the data hierarchically. The main difference from clustering is that the partitioning criterion in the decision tree is supervised with the class label in the training instances. Partitioning is simple to implement and highly interpretable and a useful application is to create a diagnostic heuristic for a disease. Given symptoms and outcomes for a population, partitioning can be used to generate a hierarchy of questions to help diagnose new patients. Predictors (split-criteria) can be either continuous or categorical (nominal or ordinal). If a predictor is continuous, then the splits are created by a cutting value. Thus, the sample is divided into values below and above the cutting one. If a predictor is categorical, then the sample is divided into two groups of levels: continuous or categorical (nominal or ordinal). If the response is continuous, then the platform fits the means of the response values. If the response is categorical, then the fitted value is a probability for the levels of the response. To properly estimate the residual uncertainty of classification, several numerical indexes are used. Among them the Gini heterogeneity index (I_G_) is calculable from relative frequencies (p_i_) of each of the M total classes [14]. Another one is the entropy (H) which is associated with the concept of quantity of uncertainty [15]. By using one of the above-mentioned indexes the algorithm is able to make a decision on which partition to make at each step of tree construction.

#### Ethics statement

The study entered a main project that was approved by local Ethical Commission and each enrolled patient gave written informed consent before enrolment (Comitato di Bioetica, Fondazione IRCCS Policlinico San Matteo, approval numbers: protocol #20090002344; procedure # 20090019080; date of approval: June 3rd, 2009).

## Results

### Data description and screening

#### Epidemiology and demographics

The total number of patients referred to our *Territorial Diagnostic, Therapeutic and Assistance Planning* was 105. From there, 17 patients did not fit our inclusion criteria. In our raw data, we looked at more than 30 parameters in 88 MPM patients between November 2018 and May 2020. Exhaustive data are summarized in Table 1. Of them, 26 (29.5%) were females and 62 (70.5%) were males. The male-to-female ratio we report is roughly 2.4, which is near to that reported in the 6th edition of the ReNaM report (M:F = 2.5) [6]. The male predominance was expected as exposure to asbestos often occurred in and around industrial factories where most employees were men. The average age at diagnosis was 68.9 years (st.dev 8.6) (from 47 to 85 years), with a median age of 71. This data is in line with that of the 6th ReNaM report, which shows an average age at diagnosis of 70 years [6]. No cases of diagnosis under the age of 47 were found in our study, confirming what is contained in the ReNaM report where only 2% of recorded cases occurred in patients younger than 45 years and coherently to the significant latency associated with the disease onset after exposure to asbestos. The majority of the observed patients had a significant exposure to asbestos as part of their employment/family/social history. Indeed, 26 out of the 88 patients reported certain or highly probable workplace exposure (either directly from the factory or indirectly through various blue-collar work). Environmental and indirect exposure cases (patients who were relatives of workers in asbestos industrial plants) were significantly higher (32 cases, 36.4%) as the pollution from the factories had an effect on members of the town. The significant environmental pollution and massive exposure during the company’s peak activity period (1970s and 1980s) created this high incidence, as previously mentioned. No relevant exposure history was reported for 30 (34%) of the patients in our cohort. Out of the 88 total patients, 36 (roughly 41%) claimed to have never smoked, while 15 (17%) were active smokers and 37 (42%) were ex-smokers. Thus, 59% of the analyzed population referred a smoking history which is coherent to previous studies. This correlation highlights the important synergistic tumorigenic activity of smoke and asbestos, as both exposures have inflammation and direct DNA damage as common underlying pathogenic mechanisms [16, 17]. Indeed, even with equal asbestos exposure, cigarette smoke has been correlated with increased risk of developing MPM [18].

**Table 1:** Exhaustive demographic and clinical data of the cohort analyzed

Features	Patients
**Gender**	
Males	62
Females	26
**Asbestos exposure**	
Work	26
Environmental	32
None	30
**Smoking habit**	
Never	36
Active smokers	15
Past smokers	37
**Radiologic findings**	
Pleural effusion	84
Bilateral alterations	34
Mediastinal involvment	34
Diaphragm involvement	45
**Diagnostic approach**	
Medical thoracoscopy	50
VATS	19
CT-guided biopsy	19
**Histology**	
Epitheliod	66
Sarcomatoid	6
Biphasic	7
NOS	9
**Disease stage (TNM)**	
I	37
II	30
IIIA	5
IIIB	9
IV	7
**Therapy**	
*Surgery*	
P/D	46
Palliative surgery	6
*Chemotherapy*	
*I line*	
Platinum-pemetrexed	67
Monochemotherapy	18
*II line*	52
Gemcitabine	30
Vinorelbine	19
Re-challenge	3
*Radiotherapy*	
Debulking	3
Palliative	4
**Outcome**	**Months**
PFS	9.8
OS	15.6

VATS: video-assisted thoracoscopy, CT: computed-tomography, NOS: not otherwise specified, TNM: tumor-nodes-metastases, PFS: progression free survival, OS: overall survival

#### Radiological and clinical diagnosis and staging algorithms

For all the 88 patients included in the analysis, we aimed at identifying key radiological features at baseline (Table 1). History of thoracentesis or drain placement was permitted as part of our selection criteria, as this would not alter plaque/tumor size. It should be noted that pleural plaques are a completely benign conditions and should not be considered as a malignant radiological feature. Even though a great number of our patients had a significant pleural effusion, the majority of them did not received thoracentesis or drainage before the “indicative” or “diagnostic” CT scan was taken; only 14 patients (16%) had evidence of a previous evacuation of pleural fluid via drain or thoracentesis on radiologic reports or CT images. On the other hand, only 4.5% (4 cases) of patients had no pleural effusion. Four patients had evidence of bilateral pleural effusion on pre-treatment CT scans. It should be noted that this finding is not surprising, and it is coherent to literature evidence [19–22], confirming that MPM does not behave like other malignancies when it comes to pleural effusions. Overall, the 96% pleural effusion prevalence reported in our study population was higher than already published data [23, 24]. For instance, Dogan et al. found a 79% prevalence in a CT analysis of 212 patients with MPM in Turkey [25], although this cohort of patients was defined by different features in terms of patient population, time-period analysis, environmental and epidemiological factors. In our study, 34 (39%) patients showed bilateral alterations (pleural effusion, pleural plaques or chest wall infiltration) whereas 64 (73%) showed mediastinal involvement at the time of diagnosis. Roughly half of our patients (45 patients or 51%) had some sort of involvement of the diaphragm before receiving chemotherapeutic treatment.

MPM diagnosis confirmation has been reached through three main approaches, namely, medical thoracoscopic, percutaneous and surgical biopsy (Table 1). Of them, medical thoracoscopy was the most common (50 patients or 56.8%). Percutaneous biopsies and surgical procedures (VATS) were used in 19 patients (21,5%) respectively, while no patient received diagnosis through cytology on pleural fluid. Biopsy results showed that most of our patients received a diagnosis of epithelioid-type MPM (66 patients or 75%). This result is in-line with the reports from AIOM 2018 guidelines that showed a prevalence of the epithelioid histotype in 75–80% of cases [26], which is significantly higher than the percentage of epithelioid cases reported in the ReNaM (55% of cases) [6]. Similar to already reported data, the epithelioid histotype was associated with an increased overall survival (15.72 months vs 13.2 months in sarcomatoid cases and 11.8 in biphasic ones). The diagnosis of a sarcomatous/desmoplastic lesion was found in 6 patients (6,8%) and the biphasic type in 7 patients (7,9%) respectively, being less represented than the data reported in ReNaM, where the biphasic histotype accounts for 10.5% of cases [6]. These percentages are also slightly different from those reported in the AIOM guidelines, where the biphasic histotype corresponds to 10–25% of cases and the sarcomatoid about 10%. In nine patients (10.2%) it was not possible to define a precise histotype even in the presence of certain MPM (defined as unspecified mesothelioma). This data is broadly in line with the ReNaM data, where 12% of MPM are not otherwise specified (NOS).

Of the 88 patients observed, 23 were stage IA, 14 patients were stage IB, 30 patients were stage II, 5 patients were classified as IIIA and 9 as stage IIIB, according to the 8th TNM version published by the International Association for the Study of Lung Cancer (IASLC) [27]. Finally, seven patients were classified as stage IV (Table 1). With respect to each component of the TNM classification, the vast majority of cases presented with a T1 mass (43 cases, 48.9%), whereas 30 cases (34.1%) featured a T2 disease, 9 (10.2%) T3 and 4 (4.5%) a T4 primary mass, respectively. The lymphnode involvement was the following: 37 cases (42%) presented with N0 and N1 disease, respectively; 10 cases (11.4%) with N2 disease and 4 (4.5%) with N3 involvement. Three cases (3.41%) presented with distant metastases (two patients carried brain and 1 case pelvis bone secondary lesions, respectively). Overall, 76% of patients have an early stage of disease (IA-B, II). As a result, most patients were susceptible to surgical therapy and/or multimodal approaches.

#### Treatments

The main therapeutic strategies for MPM are surgery, chemotherapy, and radiation therapy. The optimal precise sequence of treatments within the trimodality is unclear and should be decided upon by a multidisciplinary consensus for each individual patient [28]. It is thus clearly recommended by guidelines (AIOM [25], NCCN [29]) that MPM patients susceptible to multimodal approaches encompassing surgery, should be referred to specialized centers, and evaluated by multidisciplinary team to assess the most optimal treatment sequence in a personalized manner. In accordance with the previously described guidelines, each of the patients in our cohort was referred to surgery as the first therapeutic approach after a multidisciplinary evaluation considered clinical stage (early disease, epitheliod histotype, good performance status in absence of relevant comorbidities) and demographic parameters (age). More than half of the 88 patients in our study received some form of surgery as a treatment for MPM (52 patients or 59,1%). Of these, 46 patients received pleurectomy/decortication surgery; among them 10 patients underwent video-assisted thoracoscopic surgery (VATS). Six patients received various other palliative surgical approaches. These data correlate with the fact that most of the evaluated patients had low-stage tumors (TNM stage I or II) and were thus eligible for surgery. No significant complications and/or serious adverse events followed surgery. In all cases, P/D was followed by conventional chemotherapy and 21 patients had a progression free survival greater than 10 months. Chemotherapy was the most common therapeutic option and 67 out of the 88 patients received the standard first line chemotherapy regimen of pemetrexed and cisplatin, while 18 patients underwent mono-chemotherapy (carboplatin) due to low performance status and comorbidities. The timing of the treatment was not the same for all patients though: 4 (6%) of the 67 patients received neoadjuvant treatment and the remaining underwent adjuvant chemotherapy. Side effects of the chemotherapy were not specifically monitored, but we did encounter severe toxicities. Second line treatments were performed in 52 patients: 30 of them were treated with gemcitabine, 19 with vinorelbine and three underwent first line chemotherapy re-challenge.

Only seven patients of the entire cohort (8%) received radiotherapy (RT). In three cases RT was a part of multimodal treatment, while 4 patients received radiotherapy for palliation.

Data are detailed in Table 1.

#### Outcome

**Fig. 2:**
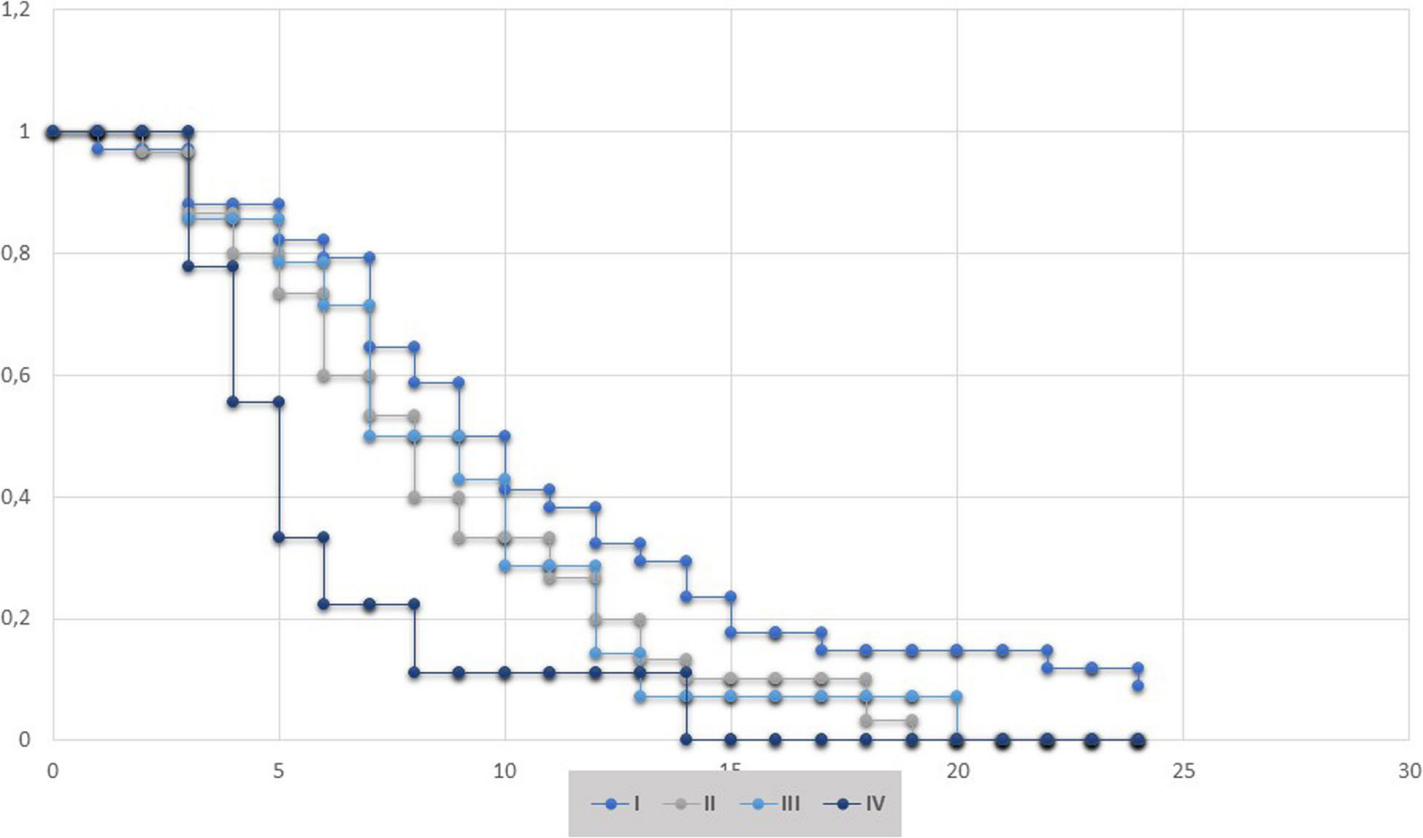
Kaplan-Meier survival curves by stage. Plot of overall survival (OS) probability (Y-axis) by TNM disease stage. Time (X-axis) is expressed in months. Crosses indicate censored patients

The number of months that a patient was “disease-free” was largely heterogeneous between 3 and 45 months with an average value of 9.8 months. The average time to progression after second line chemotherapy was 4.6 months. The average overall survival (OS) was 15.6 months (Fig. 2) irrespective of tumor histotype whereas higher OS values were observed in female patients (mean of 18.2 months vs 14.6 months in women and men, respectively).

### Statistical analysis and data mining

**Fig. 3:**
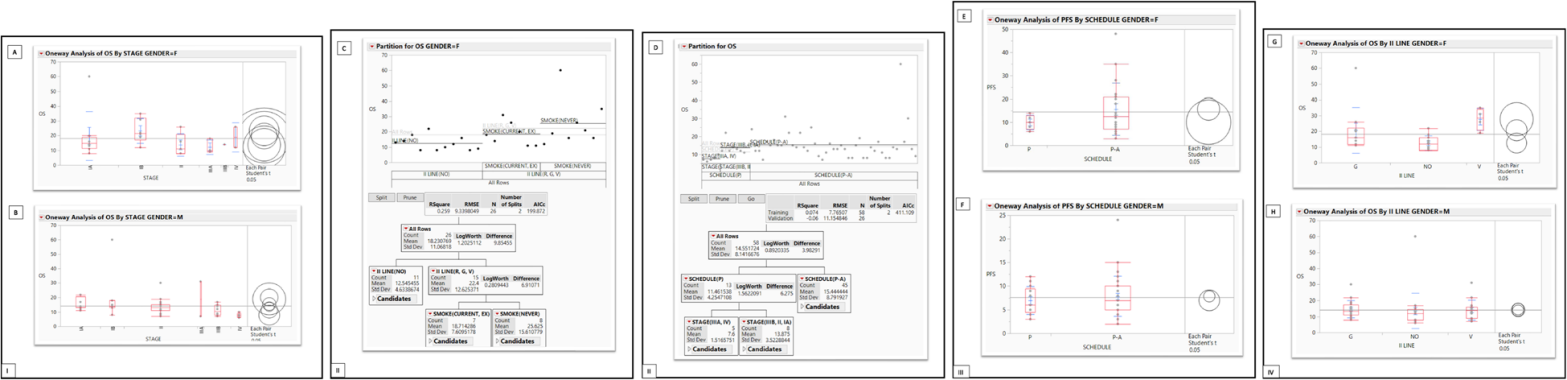
Statistical and partitioning analysis of MPM patient data. Panel I. Distribution of overall survival vs TMN by gender. Distribution of OS showed no statistically significant difference if compared to TNM disease stage, except for a very slight difference related to stage IB MPM in females (**A**) vs males (**B**). Positive values show pairs of means that are significantly different. Standard deviation error bars are shown as well. Panel II. Partition analysis for overall survival of whole data. The split criterion divides the tree through a top-down clustering algorithm. Results are represented in female (**C**) and male (**D**) subgroups, respectively. Count: number of training observation; G2: Gini index. Lower values indicate better fit. Panel III. Comparison of progression free survival vs chemotherapy schedule. Distribution of PFS showed no statistically significant difference if compared to first line chemotherapy in both females (**E**) vs males (**F**). Positive values show pairs of means that are significantly different. Standard deviation error bars are shown as well. Panel IV. Comparison of overall survival vs second line chemotherapy schedule. Distribution of OS showed moderate significant difference in female patients treated with vinorelbine vs those treated with gemcitabine (**G**); no statistically significant differences can be shown in males (**H**). Positive values show pairs of means that are significantly different. Standard deviation error bars are shown as well. OS: overall survival, PFS: progression free survival, R: first line re-challenge chemotherapy, V: vinorelbine, G: gemcitabine, P: platinum. P-A: platinum-pemetrexed, NO: second line chemotherapy not performed

We further analyzed the records through partition analysis to explore a combination of factors that could impact clinical outcome, to identify and select the most relevant predictive and prognostic variables. Differences emerged by subdividing the cohort based on patient gender. We thus focused on the most relevant outcome parameter, namely overall survival. We expected that the high number of early-stage diseases in our population study would correlate with a relatively better prognosis if compared to advanced ones, but, quite surprisingly, no statistically significant association could be identified between OS and TNM stage at diagnosis (Fig. 3, panel I). The most relevant variable associated to OS in women was determined by access to second line treatment, since those patients who did not receive it displayed a significantly lower OS (12.5 months vs 22.4 months, Fig. 3, panel II-C). Among patients who received second line therapy, the subsequent split underlined that exposure to cigarette smoke (past and/or current) significantly affected mortality (average OS 18.7 months). Although, performance status (PS) should be a limitation in defining therapeutic strategies, these data suggested that, at least in females who never smoked, comprehensive chemotherapeutic regimens can assure better outcomes (mean OS 25.6 months). Concerning males, it should be noted that no significant splits could be found when patients were stratified by age. When removing this variable from the analysis (Fig. 3, panel II-D), chemotherapy schedule significantly impacted OS and treatment with platinum and pemetrexed was associated with OS rates (15.4 months) that were higher than treatment with platinum alone (11.4 months). Coherently, concomitant advanced disease stage was associated with worse prognosis (mean OS of 5 months). Again, although PS and comorbidities might guide the decision between different treatment schedules, our study demonstrated that such decisions were irrelevant and all patients without contraindication should receive dual chemotherapy regimens. It should be noted that the results of the analysis impacted long term overall outcome and no significant differences could be found by evaluating chemotherapy regimen and progression free survival (PSF) in both men and female patients (Fig. 3, panel III). Since no clear data are available in the literature on the most beneficial conventional chemotherapy agent [30, 31], we proceeded to analyze data regarding second line treatments. Specifically, we compared the efficacy of gemcitabine (30 patients) vs vinorelbine (19 patients). We excluded from the analysis the three patients who underwent first line chemotherapy re-challenge. Based on distribution comparison a statistically significant different has been observed in OS in female patients treated with vinorelbine in second line setting (Fig. 3, panel IV-G) whereas in the male subgroup the Student’ t test comparing the pair did not reach a statistical significance. Within the limit of the cohort in analysis, this finding could be an interesting open point requiring deep investigation in dedicated works in next future.

## Discussion

Malignant pleural mesothelioma is an aggressive cancer linked to industrial pollution that causes increased death rates in correlation with areas of higher asbestos exposure, as is found in certain regions of Northern Italy. In the present study we evaluated the data from a cohort of MPMs, that was collected by the PDTA, which is dedicated to the management of this disease, with the ultimate goal of detecting and identifying predictive and prognostic variables for routine management. Preliminary findings of this work allow us to conclude the following: first, despite the male predominant occupational exposure to asbestos (and thus increased M:F ratio), no significant difference emerges between men and women regarding median age at diagnosis. This observation suggests that susceptibility to asbestos fibers is independent of gender and confirms the absence of a dose limiting cancerogenicity, since the outcome is similar in cases of higher (work) and lower (environmental/indirect) rates of exposure. Second, the stage of the tumor at diagnosis in the vast majority of cases was lower than TNM III. The main reason for the large proportion of patients diagnosed in early-stage disease can be explained by the elevated incidence in the North-Western Regions of Italy due to the asbestos-laden cement factories that were open until roughly 20 years ago. The Lombardy Region has the highest incidence of MM in Italy (18% of all cases). Within this Region, the province of Pavia (where our Institution is located and where most enrolled patients live) has by far the highest incidence of MPM due to the presence of the second oldest and largest asbestos cement factory in Italy. Asbestos cement production began in 1932 and was halted following the approval of law 257/1992 on asbestos ban [32]. Every year, more than 40 new cases of MPM are diagnosed in the province of Pavia alone, confirming that MPM in the Lombardy region represents a major public health issue. Thus, diagnosis was made early because of the increased awareness of MPM in our region and the specific diagnostic and therapeutic processes that have been implemented at a provincial and regional level. These diagnostic and therapeutic strategies serve as secondary and tertiary prevention for the community by providing prompt and comprehensive evaluation of at-risk subjects, and optimal care of affected patients.

Third, we concluded, quite unexpectedly, that there was no significant correlation between TNM disease stage and OS. Based on the partition analysis approach, this finding means that the considered variables (TNM stages) cannot predict disease outcome (OS), when conventionally treated. The multidisciplinary management of each case allowed for accurate disease staging and no serious/adverse complications in patients that underwent P/D. Thus, the observed data outline how TNM classification should be integrated with other variables (such as performance status, comorbidities, tumor histology and genetic data) to define integrated scores that can correlate with clinical outcome. Further research is needed, especially in the accelerating field of tumor genomics to allow an appropriate MPM classification and better patient stratification.

Although several limitations affect the analyzed cohort, these results confirm that the TNM staging system is likely inadequate to manage MPM alone. Furthermore, differing amounts of pleural effusion did not significantly correlate with the disease stage. One would expect that the presence of pleural effusion would correlate with more advanced stages of disease, but this did not seem to be the case, and to a small extent, the opposite seemed true.

Within the limit of the MPM population evaluated, patient gender seemed to impact outcome and OS since females displayed better average OS, especially when fully treated and in absence of exposure to cigarette smoke. Conversely, a worse prognosis was observed in males who underwent mono-chemotherapy and displayed advanced disease at diagnosis. No significant impact on OS was associated with surgery whereas no conclusions could be drawn with respect to radiotherapy due to the small number of patients treated.

## Conclusion

In conclusion, the preliminary results of this study suggest that, at least in routine settings with acceptable performance status and limited disease stage, an extensive chemotherapeutic program should be chosen to assure better outcomes. Moreover, our data showed a slight advantage of vinorelbine vs gemcitabine as second line standard chemotherapy in female patients. Thus, a multidisciplinary management of MPM should help to identify those patients who can really benefit from a more aggressive pharmacological approach. A wider set of data is needed to confirm the findings of this work and exclude a statistical fluctuation as possible explanation of the observed correlations. Overall, these results point out the high complexity of MPM, the substantial inadequacy of conventional diagnostic and therapeutic approaches, and contribute to a better understanding of the starting point for personalized therapeutic strategies.

## Supplementary Information

**Additional file 1.**

## Data Availability

Exhaustive excel database is available as supplementary material.

## Abbreviations

MPM: malignant pleural mesothelioma
PDTA: Percorso Diagnostico Terapeutico e Assistenziale- Territorial Diagnostic, Therapeutic and Assistance Planning
AIOM: Associazione Italiana di Oncologia Medica
ReNaM: Registro Nazionale Mesoteliomi
CT: computed tomography
RT: radiotherapy
CNB: core needle biopsy
P/D: pleurectomy/decortication
EPP: extrapleura pneumonectomy
VATS: video-assisted thorascopic surgery

## References

[1] Pignochino Y, Dell'Aglio C, Inghilleri S, Zorzetto M, Basiricò M, Capozzi F, Canta M, Piloni D, Cemmi F, Sangiolo D, Gammaitoni L, Soster M, Marchiò S, Pozzi E, Morbini P, Luisetti M, Aglietta M, Grignani G, Stella GM (2015). The combination of sorafenib and everolimus shows antitumor activity in preclinical models of malignant pleural mesothelioma. BMC Cancer.

[2] Røe OD, Stella GM (2015). Malignant pleural mesothelioma: history, controversy and future of a manmade epidemic. Eur Respir Rev.

[3] Berzenji L, Van Schil P. Multimodality treatment of malignant pleural mesothelioma. F1000Res. 2018 ;7: F1000 Faculty Rev-1681. doi: 10.12688/f1000research.15796.1. 10.12688/f1000research.15796.1 PMC6198256 30410726

[4] Remon J, Passiglia F, Ahn MJ, Barlesi F, Forde PM, Garon EB, Gettinger S, Goldberg SB, Herbst RS, Horn L, Kubota K, Lu S, Mezquita L, Paz-Ares L, Popat S, Schalper KA, Skoulidis F, Reck M, Adjei AA, Scagliotti GV (2020). Immune checkpoint inhibitors in thoracic malignancies: review of the existing evidence by an IASLC expert panel and recommendations. J Thorac Oncol.

[5] Nicolini F, Bocchini M, Bronte G, Delmonte A, Guidoboni M, Crinò L, Mazza M (2020). Malignant pleural mesothelioma: state-of-the-art on current therapies and promises for the future. Front Oncol.

[6] Robinson BM (2012). Malignant pleural mesothelioma: an epidemiological perspective. Ann Cardiothorac Surg.

[7] Delgermaa V, Takahashi K, Park EK, Le GV, Hara T, Sorahan T (2011). Global mesothelioma deaths reported to the World Health Organization between 1994 and 2008. Bull World Health Organ.

[8] McDonald JC, McDonald AD (1996). The epidemiology of mesothelioma in historical context. Eur Respir J.

[9] Barbiero F, Giangreco M, Pisa FE, Negro C, Bovenzi M, Rosolen V, Barbone F (2016). Standardization of incidence rates of mesothelioma in the absence of national standards: sensitivity analysis in a cohort formerly exposed to asbestos. La Medicina del lavoro.

[10] Marinaccio A, Corfiati M, Binazzi A, Di Marzio D, Scarselli A, Ferrante P, ReNaM working group (2018). The epidemiology of malignant mesothelioma in women: gender differences and modalities of asbestos exposure. Occup Environ Med.

[11] Consonni D, De Matteis S, Dallari B, Pesatori AC, Riboldi L, Mensi C (2020). Impact of an asbestos cement factory on mesothelioma incidence in a community in Italy. Environ Res.

[12] Oddone E, Bollon J, Nava CR, Minelli G, Imbriani M, Consonni D, Marinaccio A, Magnani C, Barone-Adesi F (2020). Forecast of malignant peritoneal mesothelioma mortality in Italy up to 2040. Int J Environ Res Public Health.

[13] Capecchi S, Iannario M (2016). Gini heterogeneity index for detecting uncertainty in ordinal data surveys. METRON.

[14] Eliazar I, Sokolov IM (2020). Maximization of statistical heterogeneity: from Shannon’s entropy to Gini’s index. Physica A.

[15] Abbott DM, Bortolotto C, Benvenuti S, Lancia A, Filippi AR, Stella GM (2020). Malignant pleural mesothelioma: genetic and Microenviromental heterogeneity as an unexpected Reading frame and therapeutic challenge. Cancers (Basel).

[16] Ngamwong Y, Tangamornsuksan W, Lohitnavy O, Chaiyakunapruk N, Scholfield CN, Reisfeld B, Lohitnavy M (2015). Additive synergism between Asbestos and smoking in lung Cancer risk: a systematic review and meta-analysis. PLoS One.

[17] Muscat JE, Wynder EL (1991). Cigarette smoking, Asbestos exposure, and malignant mesothelioma. Cancer Res.

[18] Segal A, Sterrett GF, Frost FA, Shilkin KB, Olsen NJ, Musk AW, Nowak AK, Robinson BW, Creaney J (2013). A diagnosis of malignant pleural mesothelioma can be made by effusion cytology: results of a 20 year audit. Pathology..

[19] Negi Y, Kuribayashi K, Funaguchi N, Doi H, Mikami K, Minami T, Takuwa T, Yokoi T, Hasegawa S, Kijima T (2018). Early-stage Clinical Characterization of Malignant Pleural Mesothelioma. In Vivo.

[20] Kato K, Gemba K, Fujimoto N, Aoe K, Takeshima Y, Inai K, Kishimoto T (2016). Pleural irregularities and mediastinal pleural involvement in early stages of malignant pleural mesothelioma and benign asbestos pleural effusion. Eur J Radiol.

[21] Hjerpe A, Abd Own S, Dobra K (2020). Integrative approach to cytologic and molecular diagnosis of malignant pleural mesothelioma. Transl Lung Cancer Res.

[22] Cardinale L, Ardissone F (2017). Diagnostic Imaging and Workup of Malignant Pleural Mesothelioma. Acta Biomed.

[23] Wang ZJ, Reddy GP, Gotway MB, Higgins CB, Jablons DM, Ramaswamy M, Hawkins RA, Webb WR (2004). Malignant pleural mesothelioma: evaluation with CT, MR imaging, and PET. Radiographics..

[24] Tamer Dogan O, Salk I, Tas F, Epozturk K, Gumus C, Akkurt I, Levent OS (2012). Thoracic computed tomography findings in malignant mesothelioma. Iran J Radiol.

[25] Associazione Italiana di Oncologia Medica (AIOM), website at https://www.aiom.it/wpcontent/uploads/2019/10/2019_LG_AIOM_Mesotelioma.pdf

[26] Berzenji L, Van Schil PE, Carp L. The eighth TNM classification for malignant pleural mesothelioma. Transl Lung Cancer Res. 2018; 7(5):543–549. doi: 10.21037/tlcr.2018.07.05 10.21037/tlcr.2018.07.05 PMC6204412 30450292

[27] Gomez D, Tsao AS (2014). Local and systemic therapies for malignant pleural mesothelioma. Curr Treat Options in Oncol.

[28] National Comprehensive Cancer Network (NCCN) website at, https://www.nccn.org/store/login/login.aspx? ReturnURL=https://www.nccn.org/professionals/physician_gls/pdf/mpm.pdf

[29] Buikhuisen WA, Hiddinga BI, Baas P, van Meerbeeck JP (2015). Second line therapy in malignant pleural mesothelioma: a systematic review. Lung Cancer.

[30] de Gooijer CJ, Baas P, Burgers JA (2018). Current chemotherapy strategies in malignant pleural mesothelioma. Transl Lung Cancer Res.

[31] Mensi C, Riboldi L, De Matteis S, Bertazzi PA, Consonni D (2015). Impact of an asbestos cement factory on mesothelioma incidence: global assessment of effects of occupational, familial, and environmental exposure. Environ Int.

[32] Scherpereel A, Opitz I, Berghmans T, Psallidas I, Glatzer M, Rigau D, Astoul P, Bölükbas S, Boyd J, Coolen J, de Bondt C, de Ruysscher D, Durieux V, Faivre-Finn C, Fennell D, Galateau-Salle F, Greillier L, Hoda MA, Klepetko W, Lacourt A, McElnay P, Maskell NA, Mutti L, Pairon JC, van Schil P, van Meerbeeck JP, Waller D, Weder W, Cardillo G, Putora PM (2020). ERS/ESTS/EACTS/ESTRO guidelines for the management of malignant pleural mesothelioma. Eur Resp J.

